# Reveal the Humidity Effect on the Phase Pure CsPbBr_3_ Single Crystals Formation at Room Temperature and Its Application for Ultrahigh Sensitive X‐Ray Detector

**DOI:** 10.1002/advs.202103482

**Published:** 2021-11-10

**Authors:** Jiayu Di, Haojin Li, Jie Su, Haidong Yuan, Zhenhua Lin, Kui Zhao, Jingjing Chang, Yue Hao

**Affiliations:** ^1^ State Key Discipline Laboratory of Wide Band Gap Semiconductor Technology School of Microelectronics Xidian University Xi'an 710071 China; ^2^ Key Laboratory of Applied Surface and Colloid Chemistry National Ministry of Education Shaanxi Key Laboratory for Advanced Energy Devices Shaanxi Engineering Lab for Advanced Energy Technology Institute for Advanced Energy Materials School of Materials Science and Engineering Shaanxi Normal University Xi'an 710119 China; ^3^ Advanced Interdisciplinary Research Center for Flexible Electronics Academy of Advanced Interdisciplinary Research Xidian University Xi'an 710071 China

**Keywords:** phase pure CsPbBr3, single crystals, solution method, ultrahigh sensitive, X‐ray detectors

## Abstract

Generally, growing phase pure CsPbBr_3_ single crystals is challenging, and CsPb_2_Br_5_ or Cs_4_PbBr_6_ by‐products are usually formed due to the different solubilities of CsBr and PbBr_2_ in the single solvent. Herein, the growth of high‐quality phase pure CsPbBr_3_ perovskite single crystals at room temperature by a humidity controlled solvent evaporation method is reported first. Meanwhile, the room temperature phase transition process from three dimensional (3D) cubic CsPbBr_3_ to two dimensional (2D) layered tetragonal CsPb_2_Br_5_ and the detailed mechanism induced by humidity are revealed. Moreover, compared with the organic–inorganic perovskite, the prepared CsPbBr_3_ single crystals are much more stable under high humidity, which satisfies the long‐term working conditions of X‐ray detectors. The X‐ray detectors based on CsPbBr_3_ single crystals show a high sensitivity and a low detection limit of 1.89 μGy_air_ s^–1^, all of which meet the needs of medical diagnosis.

## Graphical abstract

The mixed products of CsBr and PbBr_2_ are rich and diverse, which is a phenomenon of people scratching their heads. In the humidity controlled low‐temperature preparation process, the mechanism of its diversity is uncovered through the transformations of products under the influence of different conditions and a valuable guidance is provided for the subsequent crystal acquisition and growth.

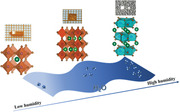

## 1 Introduction

The application of lead halide perovskites in the field of optoelectronics has attracted extensive research interest in recent years. As a new semiconductor photoelectric conversion material, it has plenty of advantages, such as high light absorption coefficient,^[^ ^1^, ^2^ ^]^ long carrier lifetime,^[^ ^3^, ^4^, ^5^, ^6^ ^]^ low defect state density^[^ ^5^ ^]^ and high carrier mobility,^[^ ^3^, ^7^ ^]^ etc. Perovskite single crystals can be prepared by the low‐temperature solution method. Compared with the traditional semiconductor preparation technology, it has the advantages of the simple fabrication method, the controllable cost and the reduced complexity of device integration.^[^ ^8^ ^]^ Moreover, single crystals of perovskites have better air stability than the thin films prepared by the spin‐coating method, which is more suitable for the long‐term application of devices.^[^ ^9^, ^10^, ^11^ ^]^

As a conversion device to convert an X‐ray photon signal into an electrical signal, the X‐ray detector is a key part of the X‐ray application system and widely used in medical diagnostic radiotherapy, industrial flaw detection, safety detection, aerospace navigation and material analysis of scientific research, et al.^[^ ^12^, ^13^ ^]^ The direct‐detection semiconductor X‐ray detector has a better sensitivity and energy resolution than the one using a scintillator.^[^ ^14^ ^]^ Generally, high‐performance direct‐detection semiconductor X‐ray detectors need to have high carrier mobility and charge carrier lifetime product for ensuring the holes and electrons have sufficient drift length, and high resistivity at room temperature.^[^ ^15^ ^]^ Furthermore, it also has a large average atomic number (Z) to ensure good absorption of radiation. In the case of certain X‐ray photon energy (E), the absorption coefficient (*α*) of X‐ray is determined by Z^4^/E^3^.^[^ ^16^ ^]^ The adjustability of elements in perovskite makes it have a large material system and also helps to adjust the average atomic number for X‐ray detection.

As for the direct detection X‐ray detector, the lead‐based perovskite semiconductor has made a series of progress due to its advantages of the high X‐ray absorption coefficient and the high carrier collection efficiency, which proves the great potential of the perovskite in the field of X‐ray detection.^[^ ^15^ ^]^ The nature of all‐inorganic perovskite CsPbBr_3_ that without the volatile organic molecules, which migrate easily under the electric field, makes its long‐term stability much enhanced.^[^ ^17^, ^18^, ^19^ ^]^ Meanwhile, the average atomic number of the all‐inorganic perovskite is larger compared to that of the organic–inorganic hybrid perovskite, which means the all‐inorganic perovskite has a higher absorption coefficient to X‐ray.^[^ ^20^ ^]^ Therefore, all‐inorganic perovskites like CsPbBr_3_ have been extensively studied for X‐ray detection.^[^ ^21^ ^]^ Amid them, the sensitivity of the X‐ray detector using CsPbBr_3_ thin film with the vertical structure of Au/film/ITO can reach 1700 µC Gy_air_^–1^ cm^–2^,^[^ ^22^ ^]^ while the highest sensitivity of the vertical Au/CsPbBr_3_ single crystal/Au X‐ray detector can reach 918 µC Gy_air_^–1^ cm^–2^.^[^ ^23^ ^]^ Though CsPbBr_3_ based X‐ray detectors have achieved much progress, most CsPbBr_3_ single crystals are grown by the high‐temperature melting method^[^ ^20^, ^24^ ^]^ and many by‐products (CsPb_2_Br_5_ or Cs_4_PbBr_6_) appear during the growth process of the low‐temperature solution method,^[^ ^25^, ^26^ ^]^ which lead to some difficulties in single crystal growth. Thus, changing the factors that affect its growth to simplify the growth method and controlling the by‐product formation is worth investigating by researchers.

In this work, we utilize a humidity controlled solvent evaporation method to synthesize various CsPbBr_3_ perovskite single crystals under the room temperature condition. The experimental results show that CsPbBr_3_ single crystals of different phases can be obtained from the precursor solution under different environmental humidity conditions. Meanwhile, phase pure transparent 2D tetragonal CsPb_2_Br_5_ single crystals could be obtained in the solution by this method under extremely high humidity conditions. While for yellow hexagonal Cs_4_PbBr_6_ single crystal (emitting green light under 365 nm light‐emitting diode (LED)), it can be prepared at the preparation temperature of 90–100°C. Finally, the planar X‐ray detector based on orthorhombic CsPbBr_3_ perovskite single crystals shows a high sensitivity of 6021.99 µC Gy_air_^–1^ cm^–2^, a low detection limit of 1.89 μGy_air_ s^–1^, all of which meet the needs of medical diagnosis. Comparing with the organic–inorganic perovskite, CsPbBr_3_ single crystals are much stable under high humidity, which is convenient for the material to satisfy the long‐term working conditions of X‐ray detectors.

## 2 Results and Discussion

**Figure 1:**
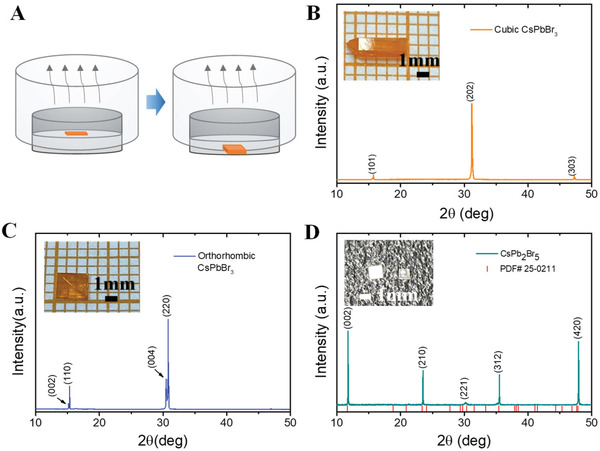
The growth model and crystal type of materials. Schematic diagrams of the growth process of CsPbBr_3_ perovskite single crystals. A) Primary stage: nucleation process due to the solution surface tension, and growth stage: single crystal sinks to the bottom of the precursors and continue to grow. B) XRD pattern of a typical cubic CsPbBr_3_ single crystal. The upper left inset is the photograph of a ≈1 × 1 × 5 mm^3^ orange CsPbBr_3_ single crystal. C) XRD pattern of the orthorhombic CsPbBr_3_ single crystal. The upper left inset is the photograph of a ≈3 × 3 × 2 mm^3^ orange CsPbBr_3_ single crystal. D) XRD pattern of the CsPb_2_Br_5_ single crystal. The upper left inset is the photograph of the transparent ≈1 × 1 × 0.3 mm^3^ CsPb_2_Br_5_ single crystal.

CsPbBr_3_ 3D perovskite single crystals were synthesized by a simple solvent evaporation method.^[^ ^11^, ^27^ ^]^ **Figure** **1**A illustrates the two stages of crystal growth during solution evaporation. Similar to the crystal growth process of other materials, the nucleation rates on both sides of the solution‐air interface are different, and the crystal nucleus is initially formed on the solution surface.^[^ ^10^, ^28^, ^29^ ^]^ When the process continues, the crystal nucleus overcomes the buoyancy, sinks to the bottom of the solution and keeps growing. After 15 d, the CsPbBr_3_ single crystal of mm‐sized was obtained. It is worth noting that the ambient humidity could affect the crystal growth behavior of CsPbBr_3_ single crystal. To investigate this strange phenomenon, XRD tests were performed on two shapes of CsPbBr_3_ single crystals. Under the condition of low humidity (RH ≈ 20%), the single crystal that grows toward the strip shape is cubic CsPbBr_3_ (the insert picture of Figure 1B), while the single crystal that grows toward the square plane (the insert picture of Figure 1C) is orthorhombic CsPbBr_3_ growing under the high humidity condition (RH ≈ 60%). Further to increases the humidity (RH > 80%), the orange CsPbBr_3_ single crystals gradually turn into opaque white crystals (shown in Figure S1A, Supporting Information). Finally, transparent square CsPb_2_Br_5_ single crystals will appear in the solution (the insert picture of Figure 1D). The same solution, which contains CsPbBr_3_ crystals, was placed in an oven with the humidity of 20% as a control experiment. However, the CsPbBr_3_ orange crystals in the control experiment are not transformed, indicating that the transformation process requires the participation of water. The opaque white crystals, on the other hand, return to orange color immediately after heating on a hot plate at 40 °C (Figure S1B, Supporting Information). Sequently removing it from the hot plate and placing it in an air environment of RH 80%, the orange color of the crystal lightens and then becomes orange and white crystals after a few days (Figure S1C, Supporting Information).

**Figure 2:**
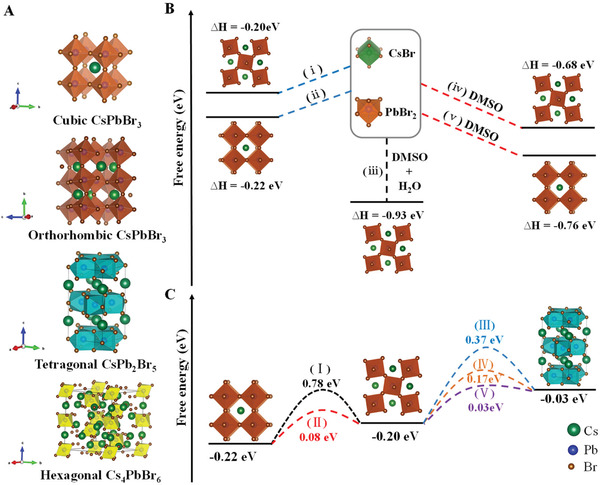
DFT calculations of phase transitions during the crystal growth. A) The unit cell structures of cubic CsPbBr_3_, orthorhombic CsPbBr_3_, tetragonal CsPb_2_Br_5_ and hexagonal Cs_4_PbBr_6_. B) The formation energies (Δ*H*) of cubic CsPbBr_3_ and orthorhombic CsPbBr_3_ under different conditions. C) The potential barriers (Δ*E*_p_) need to be overcome for the interconversion.

Figure 1B shows the X‐ray diffraction (XRD) pattern of the strip shape CsPbBr_3_ single crystal under RH ≈ 20% condition, containing a series of well‐defined periodically distributed diffraction peaks (101). Moreover, none of the other peaks are detected in the XRD patterns, which confirms its high phase purity for the fabricated cubic CsPbBr_3_. According to the thermodynamic stability properties of the phase structure reported by Stoumpos et al., the crystal structure of CsPbBr_3_ is orthorhombic at room temperature, and it could convert to tetragonal phase at 88 ℃ and cubic phase at 130 °C.^[^ ^24^ ^]^ However, the single‐crystal XRD result of orange CsPbBr_3_ shows that it belongs to a typical cubic system, with the centrosymmetric space group *Pm‐3m* (unit cell dimensions *a* = *b* = *c* = 5.857 Å, Table S1, Supporting Information for more details). This result is different from the previous cognition that cubic CsPbBr_3_ single crystals could also be grown at a low temperature. Figure 1C shows the XRD pattern of orthorhombic CsPbBr_3_ single crystals growing under RH ≈ 60% condition. Its diffraction peak is consistent with the standard card (PDF#97851), and the crystal direction is along (001) and (110) directions. The single‐crystal XRD result of square CsPbBr_3_ shows that it belongs to the orthorhombic system, with the centrosymmetric space group *Pnma* (unit cell dimensions *a* = 8.2890 Å, *b* = 11.7075 Å, *c* = 8.1066 Å, Table S1, Supporting Information for more details). Figure 1D shows the XRD pattern of transparent square single crystals growing under RH ≈ 80% condition, which is perfectly indexed to the diffraction peak of the CsPb_2_Br_5_ crystal (PDF#25‐0211). The single‐crystal XRD result of the CsPb_2_Br_5_ crystals shows that it belongs to the tetragonal system, with the centrosymmetric space group *I4/mcm* (unit cell dimensions *a* = *b* = 8.4546 Å, *c* = 15.0987 Å, Table S1, Supporting Information for more details). The corresponding unit cell structures are concluded from the single crystal XRD results and depicted in **Figure** **2**A with the sequence of cubic CsPbBr_3_, orthorhombic CsPbBr_3_, tetragonal CsPb_2_Br_5_ and hexagonal Cs_4_PbBr_6_.

In order to further explore the differences of the three structures, their band structures were calculated by Perdew–Burke–Ernzerhof functional within the generalized gradient approximation (GGA‐PBE), as shown in Figure S2 (Supporting Information). The bandgaps of cubic CsPbBr_3_, orthorhombic CsPbBr_3_ and CsPb_2_Br_5_ are 1.83, 2.07, and 3.05 eV, respectively. Besides, the three structures have similar hybrid orbits that the conduction band minimum (CBM) is contributed by Pb 5s orbit and the valence band maximum (VBM) is contributed by Br 5p orbit. Nevertheless, different from the direct band structures of the CsPbBr_3_ (cubic and orthorhombic phases), CsPb_2_Br_5_ shows an indirect band structure, which indicates a lower absorption at the wavelength of around 350 nm. There are also many reports on the crystal formation and degradation of perovskites under the influence of water.^[^ ^30^, ^31^, ^32^, ^33^ ^]^ Density functional theory (DFT) calculation is used to evaluate the formation energy to further verify the phase transition of crystal growth. The CsBr and PbBr_2_ unit cells are set as the initial system, and the total energy is 0. First, the formation energies (Δ*H*) of the cubic CsPbBr_3_ (Δ*H*_c_) and the orthorhombic CsPbBr_3_ (Δ*H*_o_) are calculated to be −0.22 and −0.20 eV, respectively. It shows that the initial system can proceed spontaneously in the direction of forming CsPbBr_3_, which corresponds to the process (i) and (ii) in Figure 2B. According to the experiments, CsBr and PbBr_2_ are dissolved in dimethyl sulfoxide (DMSO) to grow single crystals. The Δ*H*_c_ and Δ*H*_o_ that dissolved in DMSO are calculated to be −0.76 and −0.68 eV, respectively. Clearly, Δ*H*_c_ is smaller than Δ*H*_o_, indicating that the cubic CsPbBr_3_ is easier to form in DMSO. This result corresponds to processes (iv) and (v) in Figure 2B. However, when the water (H_2_O) molecule is added in this process, the Δ*H*_o_ is the lowest (−0.93 eV, corresponding to (iii)). It shows that the crystal growth process involving H_2_O could greatly affect the crystal phase, which reduces the Δ*H*_o_ of the orthorhombic CsPbBr_3_ in DMSO and promotes the reaction to proceed in this direction.

In order to explore the transformation process of orange crystals in DMSO to white opaque crystals, the conversion barriers between cubic CsPbBr_3_, orthorhombic CsPbBr_3_ and tetragonal CsPb_2_Br_5_ are further calculated by DFT. The potential barrier (Δ*E*_p_) for the direct phase transition from cubic CsPbBr_3_ to orthorhombic CsPbBr_3_ is 0.78 eV (process Ι in Figure 2C). With the participation of H_2_O, the Δ*E*_p_ is reduced to 0.08 eV (process II). The reason is that Cs^+^ and Br^–^ combine with OH^–^ and H^+^ respectively after absorbing H_2_O, thereby changing the bond length.^[^ ^31^ ^]^ Without the participation of H_2_O, two processes are involved when the orthorhombic CsPbBr_3_ transforms to tetragonal CsPb_2_Br_5_:^[^ ^34^ ^]^ one is the decomposition of CsPbBr_3_ (Δ*E*_p_ = 0.37 eV, process III), and the other is the combination of CsPbBr_3_ and PbBr_2_ in the DMSO (Δ*E*_p_ = 0.17 eV, process IV). However, with the participation of H_2_O, this process uses CsPbBr_3_ as a catalyst for the photolysis of water^[^ ^35^, ^36^, ^37^ ^]^ to produce H_3_O^+^ and OH^–^ to replace part of the Cs^+^ and Br^–^ in the crystal structure, thereby extracting hydrates and producing CsBr.^[^ ^38^ ^]^ The Δ*E*_p_ required for this process is only 0.03 eV (process V). This result also corresponds to the experimental result that no phase transition occurred in the control group in the oven. Namely, in the process of transforming CsPbBr_3_ to CsPb_2_Br_5_, it is first transformed into orthorhombic CsPbBr_3_ with the participation of H_2_O, and then into CsPb_2_Br_5_. In addition, the transparent CsPb_2_Br_5_ single crystal will not decompose like a white opaque crystal when being heated at 40–120 ℃, which can be explained by the inverse process of (III), (IV), and (V) in Figure 2C. The white opaque substance is the reverse process of (V), in which it only needs to overcome the Δ*E*_p_ of 0.03 eV (corresponding to the electron temperature of 0.026 eV at room temperature (300k)). It is easy to overcome and can occur with mild heating. The CsPb_2_Br_5_ single crystal undergoes the reverse process of (IV), and the energy required to overcome the Δ*E*_p_ is far from being achieved under heating conditions of 40–120 ℃. Therefore, the humidity and heat stability of CsPb_2_Br_5_ is good. The reaction process and formula involved in Figure 2B,C are detailed in the supporting information of the formula part.

Meanwhile, millimeter‐sized yellow single crystals could appear when the growth temperature is raised to 90–100 °C. The yellow crystal was proved to be Cs_4_PbBr_6_ by single‐crystal XRD test (Table S1, Supporting Information for more information). The Cs_4_PbBr_6_ belongs to the hexagonal system, with the centrosymmetric space group *R‐3c* (unit cell dimensions *a* = *b* = 13.685 Å, *c* = 17.279 Å). LED with a wavelength of 365 nm is used to illuminate hexagonal Cs_4_PbBr_6_ single crystal, which emits strong green light, while cubic CsPbBr_3_, orthorhombic CsPbBr_3_ and tetragonal CsPb_2_Br_5_ single crystals have no luminescence phenomenon under the same condition (shown in Figure S3, Supporting Information). CsPb_2_Br_5_ consists of inorganic octahedral layers, each of which is neutralized and separated by Cs^+^. Connectivity modes of octahedron PbI^8–^ in the inorganic layer are connected by face‐sharing. CsPb_2_Br_5_ is a 2D layered structure with good humidity and heat stability. However, the size problem of growth still needs to be solved further. Considering the small crystal size of CsPb_2_Br_5_ and the instability of the cubic CsPbBr_3_ at room temperature, a more stable orthorhombic CsPbBr_3_ single crystal at room temperature and a certain humidity is used for further device fabrication.

**Figure 3:**
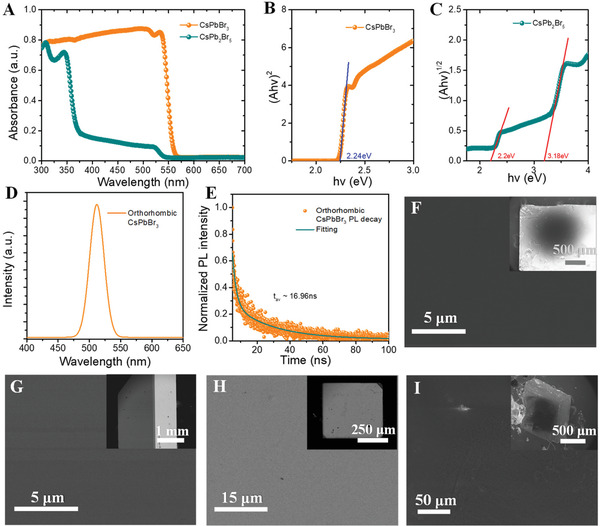
Characterizations of crystal materials. A) UV–vis absorption spectra of the orthorhombic CsPbBr_3_ powder and CsPb_2_Br_5_ powder. B) The Tauc plot for orthorhombic CsPbBr_3_. (C) The Tauc plot for CsPb_2_Br_5_. D) PL spectrum of the orthorhombic CsPbBr_3_ single crystal excited at 375 nm (FWHM = 27.4 nm). E) Time‐resolved PL (TR‐PL) spectrum of the orthorhombic CsPbBr_3_ single crystal at 512 nm. The excitation laser beam wavelength is 375 nm. F) SEM image of the orthorhombic CsPbBr_3_ single crystal with a scale bar of 5 µm. The upper right inset is the SEM image of the orthorhombic CsPbBr_3_ with a scale bar of 250 µm. G) SEM image of the cubic CsPbBr_3_ with a scale bar of 5 µm. The upper right inset is the SEM image of the cubic CsPbBr_3_ with a scale bar of 1 mm. H) SEM image of the CsPb_2_Br_5_ single crystal with a scale bar of 15 µm. The upper right insert is the SEM image of the CsPb_2_Br_5_ with a scale bar of 250 µm. I) SEM image of the Cs_4_PbBr_6_ single crystal with a scale bar of 50 µm. The upper right inset is the SEM image of the Cs_4_PbBr_6_ with a scale bar of 500 µm.

In the absorption spectrum, the absorption edge of orthorhombic CsPbBr_3_ powder is approximately at the wavelength of 553 nm, corresponding to an optical bandgap of ≈2.24 eV (**Figure** **3**A,B). Moreover, the absorption edge of CsPb_2_Br_5_ powder (white opaque crystals) is approximately at the wavelength of 390 nm, corresponding to an optical bandgap of ≈3.18 eV (Figure 3A,C). It is worth noting that CsPb_2_Br_5_ has a very low absorption edge at 563 nm, which may be due to the heat generated by grinding CsPb_2_Br_5_ crystals, resulting in its decomposition to produce CsPbBr_3_. The photoluminescence (PL) peak position of orthorhombic CsPbBr_3_ single crystal is located at 512 nm under a 375 nm laser excitation (Figure 3D), showing a blue shift relative to the absorption edge. Figure 3E shows the time‐resolved PL (TR‐PL) spectroscopy of orthorhombic CsPbBr_3_ single crystal with the same excited laser. The TR‐PL curve decays into a double exponential form, and the average carrier life (*t*_av_) is 16.96 ns, in which the fast decay lifetime (*t*_1_) and the slow decay life (*t*_2_) are 24.3 ns and 2.3 ns, respectively, accounting for 66.6% and 33.4%. The SEM images of orthorhombic CsPbBr_3_, cubic CsPbBr_3_, CsPb2Br_5_ and Cs_4_PbBr_6_ single crystals are shown in Figure 3F‐I. Figure 3G are scanning electron microscope (SEM) images of cubic CsPbBr_3_ at the scales of 5 µm and 1 mm, respectively, confirming that the CsPbBr_3_ single crystals grown by the solvent evaporation method at the low temperature have high quality. The size of the whole CsPb_2_Br_5_ crystal is small, but its quality is better, as shown in SEM (Figure 3H).

**Figure 4:**
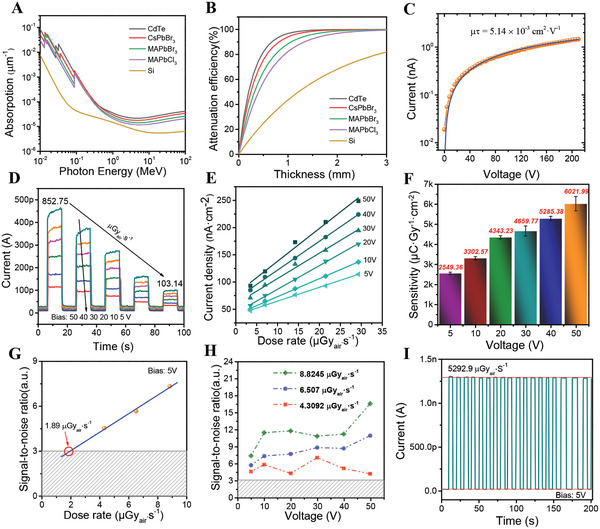
Electrical tests of the orthorhombic CsPbBr_3_ single‐crystal X‐ray Detector. A) Absorption coefficients of CsPbBr_3_, CdTe, MAPbBr_3_, MAPbCl_3_, and silicon as a function of photon energy. B) Attenuation efficiency of CsPbBr_3_, CdTe, MAPbBr_3_, MAPbCl_3,_ and silicon for 40 keV X‐ray photons versus thickness. C) Photoconductivity measurement of the CsPbBr_3_ single crystal device. D) ON/OFF current responses under various bias voltages (from 50 to 5 V) and dose rates (from 852.75 to 103.14 μGy_air_ s^–1^) of the CsPbBr_3_ single crystal X‐ray detector. E) X‐ray generated current density versus dose rate under different bias voltages. F) X‐ray sensitivity of the CsPbBr_3_ single‐crystal X‐ray detector as a function of applied voltage. G) Signal to noise ratio under different X‐ray dose rates of the CsPbBr_3_ single crystal X‐ray detector (5 V bias voltage). H) The signal‐to‐noise ratio (SNR) of CsPbBr_3_ single‐crystal device under different bias voltages and dose rates (4.3092, 6.507, and 8.8245 μGy_air_ s^–1^, respectively). I) CsPbBr_3_ single‐crystal device responses to X‐ray when turning the X‐ray source on and off. The voltage bias is 5V and the dose rate is 5292.9 μGy_air_ s^–1^.

In general, the higher the average atomic number of a material, the more X‐ray absorbability it has. The X‐ray absorption coefficients of CsPbBr_3_ and several other common semiconductor materials for photons with energy from 10 keV to 10 MeV were calculated by the NIST X‐COM application. It can be seen from **Figure** **4**A that CsPbBr_3_ has absorption coefficient much higher than that of MAPbBr_3_, MAPbCl_3_ and silicon, indicating that CsPbBr_3_ has better X‐ray detection capability, although it absorbs fewer X‐rays at about 26–90 keV than commercially available CdTe. Figure 4B is used to determine the thickness of CsPbBr_3_ single crystal applied to the X‐ray detection of 40 KeV, and it shows that when the crystal thickness reaches 1.7 mm, more than 99% of the X‐ray photons can be absorbed. The carrier mobility and mean lifetime (*µτ*) product is also one of the factors affecting the X‐ray detection capability of devices, and the value reflects the electrons extraction ability of materials. By using the modified Hecht equation (supporting information equation 1), the photoconductivity curve of CsPbBr_3_ single‐crystal vertical device can be fitted that its *µτ* product value is about 5.14 × 10^–3^ cm^2^·V^–1^, as shown in Figure 4C. This value can reach the level of CsPbBr_3_ single crystals prepared by melt growth method, which reflects the high quality of orthorhombic CsPbBr_3_ single crystal grown by low‐temperature solvent evaporation method is realized.^[^ ^20^, ^24^ ^]^ The planar device structure is utilized for the measurement to obtain the X‐ray response characteristics. Figure S4A (Supporting Information) is the *I*_dark_ *–V* diagram of reverse sweep from 50 to −50 V in the dark state of the device. The *I*_dark_ *–t* diagram of the device under 5 V bias shows that the *I*_dark_ of the device does not drift with the time change, and its value is stable around 21 pA, as shown in Figure S4B (Supporting Information). Figure S5 (Supporting Information) shows the *I–V* curves of the CsPbBr_3_ single‐crystal X‐ray detector measured with different dose rates from 103.14 to 5293 μGy_air_ s^−1^. The *I–V* curves of the device are smooth under different X‐ray dose rate irradiations and increase gradually with the augments of X‐ray dose rate (with a positive correlation).

Detection sensitivity is one of the important parameters to evaluate the performance of an X‐ray detector. Under the condition of the same X‐ray radiation dose rate, the higher the gain current density of the device means the higher detection sensitivity and the better device performance. Figure 4D shows the ON/OFF current responses of the CsPbBr_3_ single‐crystal X‐ray detector under different voltage biases (from 50 to 5 V) and dose rates (from 852.75 to 103.14 Gy_air_ s^–1^) to explore the device sensitivity. The ON/OFF current response of CsPbBr_3_ single crystal devices at 50 V bias voltage is shown in Figure S6 (Supporting Information). The dose rate is changing from 50.364 to 419.85 μGy_air_ s^–1^. The device continues to switch at 50 V high voltage, maintaining good switching characteristics. The relationship between the gain current density and the radiation dose rate of the CsPbBr_3_ single‐crystal X‐ray detector, tested at 5, 10, 20, 30, 40, 50 V bias voltage, is shown in Figure 4E. It can be clearly seen that there is an approximately linear relationship between the gain current density of the device and the X‐ray dose rate. According to the slope of the curve, the detection sensitivity of the CsPbBr_3_ single‐crystal X‐ray detector device at a low voltage bias of 5 V can reach 2549.36 µC Gy_air_^–1^ cm^–2^. When the applied voltage is increased to 50 V, the sensitivity can reach 6021.99 µC Gy_air_^–1^ cm^–2^. The high sensitivity of CsPbBr_3_ is not only related to the structural symmetry of lead‐based perovskite itself, but also due to the unique electronic configuration ns^2^np^0^ of Pb^2+^.^[^ ^39^ ^]^ Furthermore, compared with vertical or horizontal structure devices based on different materials, this parameter should be among the highest values (**Table** **1** is supported for summary comparisons). In addition, the detection sensitivity of the devices at different voltages is shown in Figure 4F.

**Table 1:** Comparison of the X‐ray detector sensitivity of various materials and device structure response to X‐rays

Materials	Device structure	Voltage bias/electric field	Sensitivity (µC Gy_air_^–1^ cm^–2^)	Lowest detectable X‐ray dose rate	Reference
*α*‐Se	Vertical	10 000 V/mm	20	‐	^[^ ^45^ ^]^
*α*‐Se	simple coplanar “p‐i‐n” Se diode	16 000 V/mm	440	‐	^[^ ^46^ ^]^
Cd(Zn)Te	Au/crystals/Au vertical	17 V/mm	1600	‐	^[^ ^47^ ^]^
Diamond	Au/Pt/SC film/Pt/Au vertical	6 V/mm	750		^[^ ^48^ ^]^
P3HT:PCBM with GOS:Tb	Al/material/TFB/ITO/a‐Si:H vertical	1000 V/mm	7.36	‐	^[^ ^49^ ^]^
MAPbBr_3_	Au/BCP/C_60_/SC/Si vertical	0.5 V/mm	322	0.036 μGy_air_ s^–1^	^[^ ^50^ ^]^
MAPbBr_3_	Au/SC/C_60_/BCP/Au vertical	10^4^ V/mm	80	0.5 μGy_air_ s^–1^	^[^ ^51^ ^]^
MAPbBr_3_	Au/SC/Au vertical	200 V	62	‐	^[^ ^41^ ^]^
MAPbBr_3_	Au/SC/Al vertical	200 V	359	‐	
MAPbBr_3_	Au/SC/Au vertical	15 V/mm	276	‐	^[^ ^21^ ^]^
MAPbBr_3_	Au/SC/MoO_3_/Au vertical	15 V/mm	786	1.2 μGy_air_ s^–1^	
MAPbI_3_	Ag/ZnO/PCBM/sintered wafer/PEDOT:PSS/ITO/Glass vertical	200V/mm	2527	0.048 mGy_air_ s^–1^	^[^ ^52^ ^]^
Cs_2_AgBiBr_6_	Au/SC/Au vertical	25 V/mm	105	59.7 nGy_air_ s^–1^	^[^ ^40^ ^]^
Cs_2_AgBiBr_6_	Ag/SC/Ag vertical	6 V/mm	316	‐	^[^ ^53^ ^]^
Cs_2_AgBiBr_6_	Au/SC/Au vertical	50 V/mm	1974	45.7 nGy_air_ s^–1^	^[^ ^54^ ^]^
MA_3_Bi_2_I_9_	Au/SC/Au horizontal	60 V/mm	1947	83 nGy_air_ s^–1^	^[^ ^55^ ^]^
Cs_3_Bi_2_I_9_	Au/SC/Au horizontal	50 V/mm	1652.3	130 nGy_air_ s^–1^	^[^ ^16^ ^]^
CsPbBr_3_	Au/film/ITO vertical	110 V/mm	1700	0.053 μGy_air_ s^–1^	^[^ ^22^ ^]^
CsPbBr_3_	Au/SC/Au vertical	15 V/cm	93	‐	^[^ ^21^ ^]^
CsPbBr_3_	Au/SC/MoO_3_/Au vertical	15 V/cm	221	20.9 μGy_air_ s^–1^	
CsPbBr_3_	Au/SC/Au vertical	30V	918	‐	^[^ ^23^ ^]^
CsPbBr_3_	Au/SC/Au horizontal	5 V/mm	2549.36	1.89 μGy_air_ s^–1^	This work

*SC is the abbreviation of single crystal.

The X‐ray radiation can cause radiation damage to creatures. Therefore, another requirement for commercial X‐ray detectors is to pursue extremely low detection limits to reduce radiation loss. Many pieces of literature employ signal‐to‐noise ratio (SNR) = 3 recommended by IUPAC as the signal detection limit.^[^ ^40^, ^41^, ^42^ ^]^ Based on this standard, SNR can reach above the detection limit at different voltages and different dose rates when testing the CsPbBr_3_ single‐crystal X‐ray detector, as shown in Figure 4G. Figure 4H shows the SNR of devices with a particular 5 V bias and different dose rates. It is difficult to measure at a lower dose rate. The detection limit of the device can reach 1.89 μGy_air_ s^–1^ by extending the fitting curve to the direction of low dose rate. This value for CsPbBr_3_ single‐crystal plane X‐ray detector is only one‐tenth of that of the CsPbBr_3_ vertical X‐ray detector,^[^ ^21^ ^]^ which is also lower than the value required for regular medical diagnostics (5.5 μGy_air_ s^–1^).^[^ ^43^, ^44^ ^]^ (A comparison of the lowest dose rates of different devices is provided in Table 1.) As is known to all, humidity, oxygen and other factors could affect the stability of perovskite materials and devices. Light also causes instability of perovskite materials, which influences the service life and performance stability of devices. Figure 4I presents the radiation response of a CsPbBr_3_ X‐ray detector under 5 V bias voltage in ambient air with *I*_on_ and *I*_off_ corresponding to ≈1.3 nA and ≈20 pA, respectively. It indicates that the device has a large ON/OFF ratio (≈50) and a steady current under a fast switching test at low bias voltage (5 V) and high dose rate (5292.9 μGy_air_ s^–1^). Figure S7 (Supporting Information) shows the CsPbBr_3_ single‐crystal X‐ray detector has good operating stability and current remains steady at 5293.9 μGy_air_ s^–1^ with ± 5 V bias voltage. These two figures show that the device performance is not degraded and still maintains good performance under high‐dose X‐ray irradiation. To sum up, CsPbBr_3_ is a promising perovskite material for X‐ray detectors.

## 3 Conclusion

High‐quality 3D CsPbBr_3_ perovskite single crystals were synthesized by a simple solvent evaporation method at room temperature. Under high humidity (80%) condition, 3D cubic CsPbBr_3_ perovskite can be transformed into CsPb_2_Br_5_ hydrate during growth, which is thermally unstable. 2D tetragonal CsPb_2_Br_5_ single crystal grown directly under RH 80% condition has good humidity and heat stability. Yellow hexagonal Cs_4_PbBr_6_ single crystal (emitting green light under 365nm LED) can be prepared at the preparation temperature of 90–100 °C. Most importantly, the planar‐structured CsPbBr_3_ perovskite single crystal X‐ray detector shows high sensitivity (more than 300 times higher than the state‐of‐the‐art commercial *α*‐Se vertical counterparts) and a low detection limit of 1.89 μGy_air_ s^–1^ (need for medical diagnosis is below 5.5 μGy_air_ s^–1^). Compare with the organic–inorganic perovskite, CsPbBr_3_ single crystals are still not decomposed under high humidity and have better air stability, which is convenient for the material to satisfy the long‐term use conditions of X‐ray detectors.

## 4 Experimental Section

### CsPbBr_3_ Single Crystal Synthesis

CsPbBr_3_ 3D perovskite single crystals were synthesized by a simple solvent evaporation method. 1.5975g CsBr (Advanced Electron Technology Co., Ltd, 99.999%) and 2.7525g PbBr_2_ (Advanced Electron Technology Co., Ltd, 99.9%) were dissolved in 30 ml DMSO (Sigma‐Aldrich, 99.9%, anhydrous) at a molar ratio of 1:1 to generate 0.25 m CsPbBr_3_ precursor. The precursors were stirred constantly in the atmospheric environment for 24 h at room temperature (25 °C). After filtration with a 0.45 µm organic filter head, the precursor solution was clarified and transferred to a precleaned crystallizing dish. The crystallizing dish was covered to prevent dust or impurities from falling contamination and to reduce the evaporation rate of the solution to obtain higher quality crystals. After that, the whole unit was placed in the oven and kept at 27 °C. Several days later, CsPbBr_3_ 3D perovskite single crystals were obtained with a size of a few millimeters. The cubic CsPbBr_3_ single crystals (tended to be a long strip) were grown in the low humidity growing environment. However, with increasing humidity, the growth tended to be an orthorhombic phase crystal structure (growing along a square plane).

### Material Characterizations

XRD was performed with a Bruker D8 Discover X‐ray diffractometer with a conventional Cu target X‐ray tube set to 40 kV and 40 mA. Cubic CsPbBr_3_, orthorhombic CsPbBr_3_, CsPb_2_Br_5_, and Cs_4_PbBr_6_ single‐crystal XRD measurements were performed with Bruker D8 Venture with Mo K*α* X‐rays. SEM images of cubic CsPbBr_3_, orthorhombic CsPbBr_3_, CsPb_2_Br_5_, and Cs_4_PbBr_6_ were collected with a tungsten filament scanning electron microscope (HITACHI SU‐3500). Absorption spectra were measured using a Perkin–Elmer Lambda 950 UV–vis–NIR spectrophotometer. Steady‐state and time‐resolved PL measurements of cubic CsPbBr_3_ and orthorhombic CsPbBr_3_ were taken using a PicoQuant FT‐300 and FT‐100, with 375 nm excitation wavelength. All material characterizations were measured in the air without encapsulation.

### Device Fabrication and Characterization

The orthorhombic CsPbBr_3_ grown in a planar shape was simpler than the cubic CsPbBr_3_ grown in a stripe shape to make a planar X‐ray detector electrode. The X‐ray detector prepared later used the orthorhombic CsPbBr_3_. Two kinds of X‐ray devices were prepared by gold electrodes deposition on orthorhombic CsPbBr_3_ perovskite single crystal for testing. Au/CsPbBr_3_/Au vertical structure devices (Au thickness is 100 nm, effective area of the device is 0.07 cm^2^ and crystal thickness is 2 mm, device structure diagram is as shown in Figure S8A, Supporting Information) measured *µτ* and horizontal structure devices (CsPbBr_3_ is 3 × 3 × 2 mm^3^, Au is 100 nm, effective area of the interpolating device is 0.012 mm^2^, device structure diagram is as shown in Figure S8B, Supporting Information) measured the X‐ray response characterization. Tungsten anode X‐ray tube (DX‐DS2901/24) was used as the source. A Keysight B2902A source table provided the bias voltage and recorded the response current. The X‐ray source operated at a constant voltage of 40 kV. The current was adjusted from 40 to 5 mA to tune the dose rate of the emitted X‐rays. Several pieces of 2 mm thick aluminum foils were inserted between the source and the CsPbBr_3_ single‐crystal X‐ray detector as attenuators. The X‐ray dose rate was carefully measured using the Fluke Si diode (RaySafe X2 R/F) dosimeter. All X‐ray response characterizations were performed directly in the dark air with optical and electrical shielding to reduce electromagnetic and ambient light interference. All measurements were performed at room temperature.

### First‐Principles Calculation

All calculations were carried out by using density functional theory based on the projector‐augmented wave method implemented in the VASP code.^[^ ^56^ ^]^ The exchange‐correction functional was described by the Generalized Gradient Approximation with the Perdew–Burke–Ernzerhof functional.^[^ ^57^ ^]^ The transformation barriers were calculated using the climbing image nudged elastic band (CI‐NEB) method through the VTST tools.^[^ ^58^, ^59^ ^]^ The plane‐wave cutoff energy was set to be 400 eV. The Monkhorst−Pack *k*‐point mesh is sampled with a separation of 0.05 and 0.015 Å^−1^ in the Brillouin zone. All structures were relaxed until the residual force on each atom was less than 0.01 eV Å^−1^. The self‐consistent convergence accuracy was set at 10^–5^ eV per atom in the structural calculation, and 10^–7^ eV per atom in the CI‐NEB calculation. The formation energies were obtained from the total energy variations of the chemical processes listed in the Supporting Information.

## Conflict of Interest

The authors declare no conflict of interest.

## Supporting information

Supporting Information

## Data Availability

The data that support the findings of this study are available in the manuscript and Supporting Information of this article.
