# Supplementary material for: Reveal the Humidity Effect on the Phase Pure CsPbBr3 Single Crystals Formation at Room Temperature and Its Application for Ultrahigh Sensitive X‐Ray Detector
Source: Adv Sci (Weinh). 2021 Nov 10;9(2):2103482. doi: 10.1002/advs.202103482 (PMC8805584; doi:10.1002/advs.202103482)
Supplement: Supplementary file 1 — Supporting Information [file ADVS-9-2103482-s001.pdf]

ADVANCED SCIENCE
Open Access

Supporting Information

for *Adv. Sci.*, DOI: 10.1002/advs.202103482

Reveal the Humidity Effect on the Phase Pure $CsPbBr_3$ Single Crystals Formation at Room Temperature and Its Application for Ultrahigh Sensitive X-ray Detector

Jiayu Di, Haojin Li, Jie Su, Haidong Yuan, Zhenhua Lin, Kui Zhao, Jingjing Chang, Yue Hao*

Supporting Information

Reveal the Humidity Effect on the Phase Pure $CsPbBr_3$ Single Crystals Formation at Room Temperature and Its Application for Ultrahigh Sensitive X-ray Detector

Jiayu Di[a]†, Haojin Li[c]†, Jie Su[a], Haidong Yuan[a], Zhenhua Lin[a], Kui Zhao[c], Jingjing Chang[a,b], Yue Hao[a]*

†These authors contributed equally to this work.

[a]State Key Discipline Laboratory of Wide Band Gap Semiconductor Technology, School of Microelectronics, Xidian University, Xi'an, China.

[b]Advanced Interdisciplinary Research Center for Flexible Electronics, Academy of Advanced Interdisciplinary Research, Xidian University, Xi'an, China.

[c]Key Laboratory of Applied Surface and Colloid Chemistry, National Ministry of Education; Shaanxi Key Laboratory for Advanced Energy Devices; Shaanxi Engineering Lab for Advanced Energy Technology; Institute for Advanced Energy Materials; School of Materials Science and Engineering, Shaanxi Normal University, Xi'an 710119, China.
E-mail: jjingchang@xidian.edu.cn

1. CsBr and $PbBr_2$ system generates cubic $CsPbBr_3$, orthogonal $CsPbBr_3$ and tetragonal $CsPb_2Br_5$ process in Figure 2a:

(i)

$$PbBr_2 + CsBr \longrightarrow CsPbBr_3 \text{ (orthorhombic phase)}, \Delta H = -0.20eV$$

(ii)

$$PbBr_2 + CsBr \longrightarrow CsPbBr_3 \text{ (cubic phase)}, \Delta H = \text{-}0.22eV$$

(iii)

$$PbBr_2 \cdot DMSO + 3PbBr_2 \cdot 2H_2O + CsBr \longrightarrow CsPbBr_3 \text{ (orthorhombic phase)}, \Delta H = \text{-}0.93eV$$

(iv)

$$PbBr_2 \cdot 2DMSO + CsBr \longrightarrow CsPbBr_3 \text{ (orthorhombic phase)}, \Delta H = -0.68eV$$

(v)

$$PbBr_2 \cdot 2DMSO + CsBr \longrightarrow CsPbBr_3 \text{ (cubic phase)}, \Delta H = -0.76eV$$

2. The process of transition from $CsPbBr_3$ to $CsPb_2Br_5$:

(I)

$$CsPbBr_3 \text{ (cubic phase)} \longrightarrow CsPbBr_3 \text{ (orthorhombic phase)}, \Delta E_p = 0.78eV$$

(II)

$$CsPbBr_3 \text{ (cubic phase)} + H_2O \longrightarrow CsPbBr_3 \text{ (orthorhombic phase)}, \Delta E_p = 0.08eV$$

(III)

$$2CsPbBr_3 \longrightarrow CsPb_2Br_5 + CsBr, \Delta E_p = 0.37eV$$

(IV)

$$CsPbBr_3 + PbBr_2 \longrightarrow CsPb_2Br_5, \Delta E_p = 0.17eV$$

(V)

$$2CsPbBr_3 + H_2O \longrightarrow CsPb_2Br_5 + CsBr \cdot H_2O, \Delta Ep = 0.03eV$$

3. Modified Hecht equation:

$$I = \frac{I_0 \mu \tau V}{D^2} \frac{1 - \exp(-\frac{D^2}{\mu \tau V})}{1 + \frac{Ds}{V \mu}} \qquad (1)$$

where I_0 is the saturated photocurrent, D is the sample thickness, μ is the carrier mobility, τ is the carrier lifetime, s is the surface composite rate, V is the voltage bias.

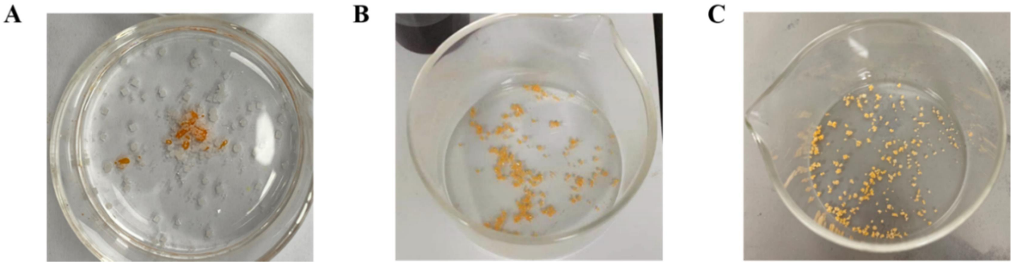

Figure S1. (A) Photograph of orange crystals gradually turning into a white mixture under high humidity conditions (RH ~ 80 %). (B) Photograph of orange crystals, which colors are white before heating. (C) Photograph of mixtures of orange and white crystals.

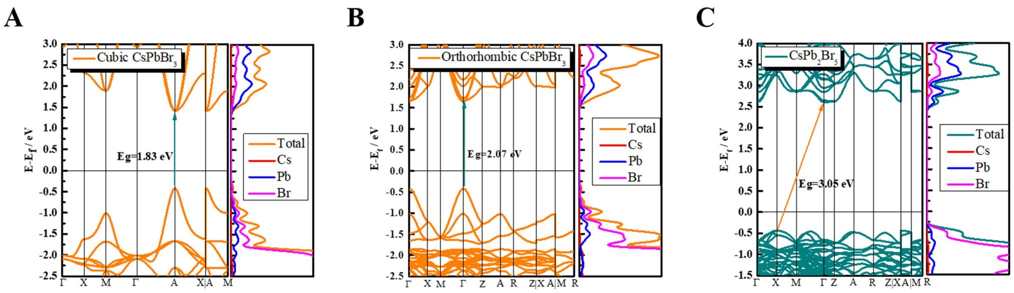

Figure S2. (A) Band structure (left) and density of states (DOS) (right) of cubic $CsPbBr_3$ calculated by GGA - PBE. (B) Band structure (left) and density of states (DOS) (right) of orthorhombic $CsPbBr_3$ calculated by GGA - PBE. (C) Band structure (left) and DOS (right) of $CsPb_2Br_5$ calculated by GGA - PBE.

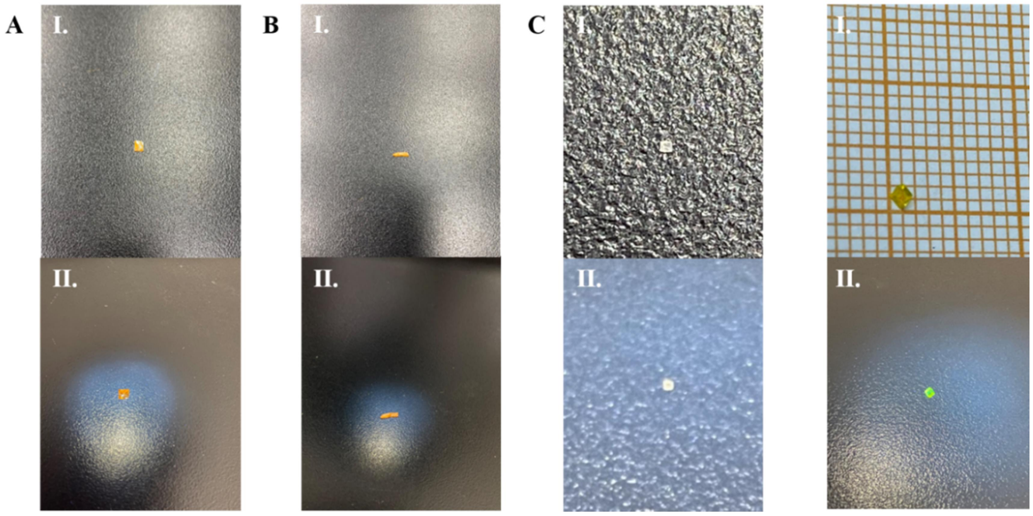

Figure S3. (A) Photograph of the orthorhombic $CsPbBr_3$ single crystal: I. under ambient light and II. under 365 nm LED light. (B) Photograph of the cubic $CsPbBr_3$ single crystal: I. under ambient light and II. under 365 nm LED light. (C) Photograph of tetragonal $CsPb_2Br_5$ single crystal: I. under ambient light and II. under 365 nm LED light. (D) Photograph of hexagonal Cs_4PbBr_6 single crystal: I. under ambient light and II. under 365 nm LED light.

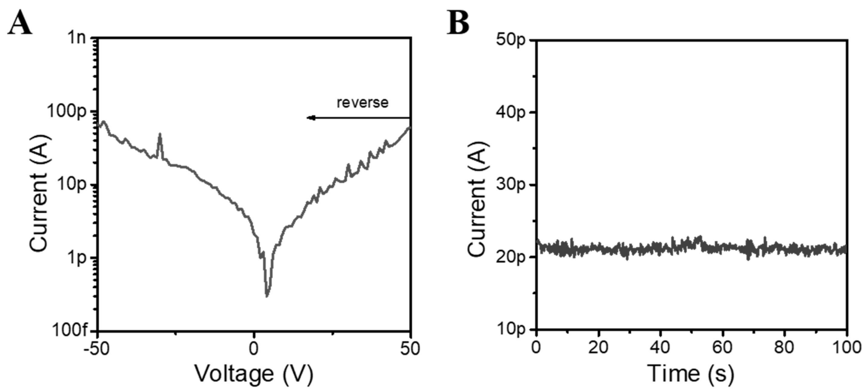

Figure S4. (A) Dark *I–V* curve of the $CsPbBr_3$ single crystal X-ray detector with reverse sweep from -50 to 50 V. (B) Dark current – Time ($I_{dark} - t$) curve of the $CsPbBr_3$ single crystal X-ray detector (5 V bias).

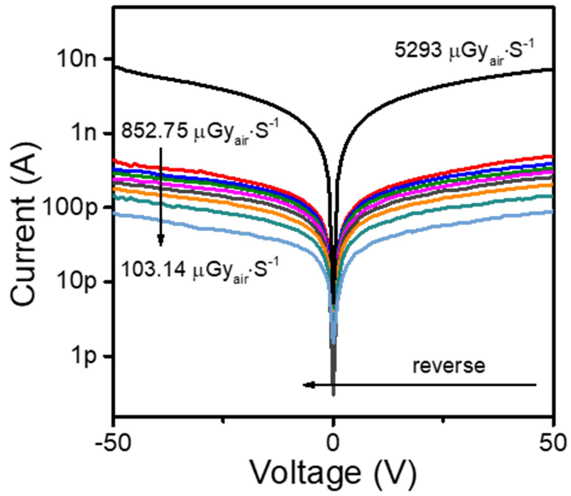

Figure S5. *I – V* curves of the $CsPbBr_3$ single crystal X-ray detector measured with dose rate from 103.14 to 5293 μGy_{air}·S^{-1}.

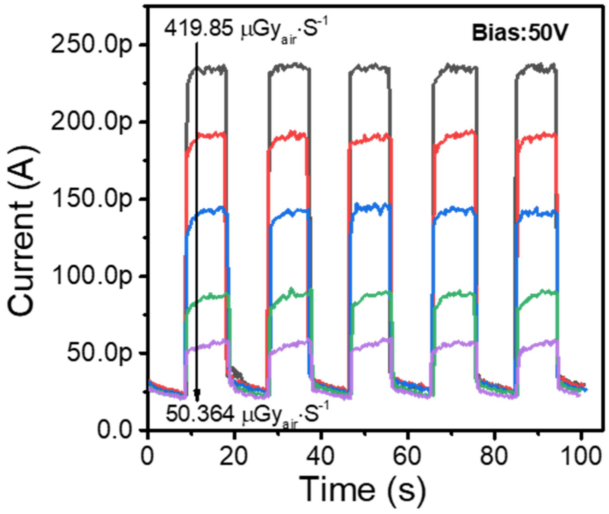

Figure S6. ON/OFF current response of $CsPbBr_3$ single crystal devices at 50 V bias voltage. The dose rate is from 50.364 to 419.85 $\mu Gy_{air}\cdot S^{-1}$.

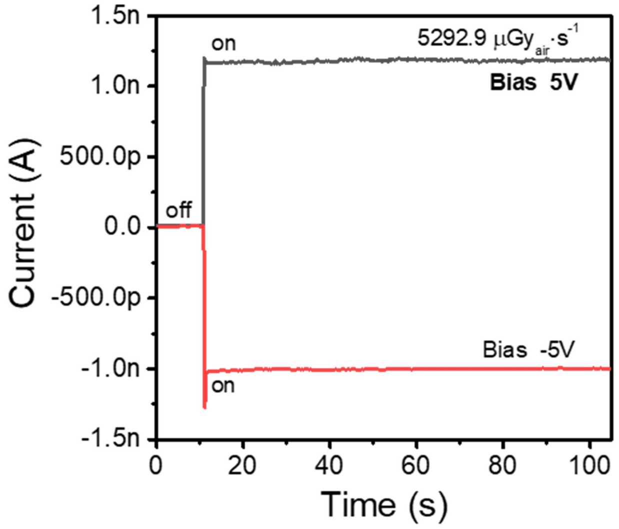

Figure S7. $CsPbBr_3$ single-crystal X-ray detector operating stability at 5293.9 $\mu Gy_{air}\cdot S^{-1}$ with ± 5 V voltage bias, tested in ambient air without any encapsulation.

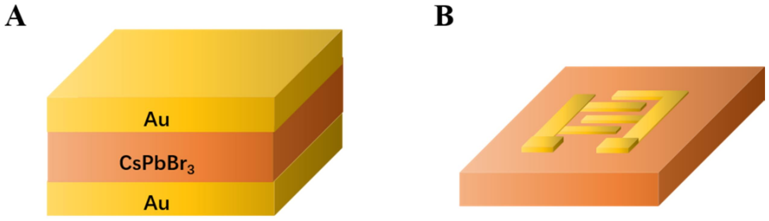

Figure S8. Schematic device structure of the $CsPbBr_3$ single-crystal X-ray detectors: (a) vertical structure, Au is 100 nm, the effective area of the device is 0.07 cm^2 and crystal thickness is 2 mm, and (b) planar structure, $CsPbBr_3$ is $3 \times 3 \times 2$ mm^3, Au is 100 nm, and the effective area of the interpolating device is 0.012 mm^2.

Table S1.

X-ray diffraction data of $CsPbBr_3$ and $CsPb_2Br_5$ single crystal.

Chemical formula	$CsPbBr_3$		$CsPb_2Br_5$	Cs_4PbBr_6
	Cubic	Orthorhombic		
Formula weight	581.25	1159.62	1893.62	1218.24
Temperature	296 k	193k	273k	273 k
Wavelength	0.71073	1.34139	0.71073	0.71073
Crystal system	Cubic	Orthorhombic	Tetragonal	Hexagonal
Space group	*Pm-3m*	*Pnma*	*I4/mcm*	*R-3c*
a	5.857(1) Å	8.2890	8.4546(5) Å	13.685(7) Å
b	5.857(1) Å	11.7075	8.4546(5) Å	13.685(7) Å
c	5.857(1) Å	8.1066	15.0987(13) Å	17.279(7) Å
α	90°	90°	90°	90°
β	90°	90°	90°	90°
γ	90°	90°	90°	120°
Volume	200.92(10) $Å^3$	786.69(8) $Å^3$	1079.26(16) $Å^3$	2803(3) $Å^3$
Z	1	2	2	6
Density(calculated)	4.08 g/cm^3	4.896 g/cm^3	5.827 g/cm^3	4.330 g/cm^3
Absorption coefficient	40.344 mm^{-1}	63.791 mm^{-1}	52.918 mm^{-1}	29.501 mm^{-1}
Theta max	30.506°	53.907°	26.963°	24.959°
Crystal size	1 × 1 × 4 mm^3	3 × 3 × 1 mm^3	1 × 1 × 0.5 mm^3	0.5× 0.5 × 0.5 mm^3
F000	242.6	968.0	1576.0	3072.0
Reflection collected	90	751	341	553
Absorption correction	0.065-0.266	0.003-0.080	0.037-0.171	0.017-0.100
Absorption correction type	Multi-scan	Multi-scan	Multi-scan	Multi-scan
